# ﻿Two new species of *Salvia* (Lamiaceae) from the dry forests of Dominican Republic

**DOI:** 10.3897/phytokeys.249.137556

**Published:** 2024-12-05

**Authors:** Itzi Fragoso-Martínez, Gerardo A. Salazar, Emmanuel Martínez-Ambriz, Martin Reith

**Affiliations:** 1 Red de Biodiversidad y Sistemática, Instituto de Ecología, A.C., Carretera Antigua a Coatepec 351, El Haya, 91073, Xalapa, Veracruz, Mexico Red de Biodiversidad y Sistemática, Instituto de Ecología Xalapa Mexico; 2 Departamento de Botánica, Instituto de Biología, Universidad Nacional Autónoma de México, Apartado Postal 70–367, 04510 Coyoacán, Mexico City, Mexico Universidad Nacional Autónoma de México México DF Mexico; 3 Jardín Botánico Prof. Eugenio de Js Marcano F., Av. del Botánico Dr. José de Jesús Jiménez Almonte 1, Jacagua, Santiago de los Caballeros, Dominican Republic Jardín Botánico Prof. Eugenio de Js Marcano F. Santiago de los Caballeros Dominican Republic

**Keywords:** Antilles, endemism, Hispaniola, phylogeny, sages

## Abstract

We describe two new species of *Salvia* from the Antillean dry forests, belonging to SalviasectionUrbania. These species’ names honor two Latin American botanists who have advanced our understanding either of the Dominican flora or the mint family (Lamiaceae). *Salviaclaseana* is found in the Sierra Martín García. It resembles *S.calaminthifolia* but differs in having strigose stems, rhombic to trullate leaves with a cuneate, decurrent base, and larger flowers. *Salviamartineziana* inhabits the Sierra de Bahoruco. It resembles *Salviabrachyphylla* but differs in having strigose stems with retrorse trichomes and flowers disposed in the axils of the distal leaves. We provide descriptions, photographs, a distribution map and an identification key for the species of SalviasectionUrbania from the Domini­can Republic. Additionally, we sequenced three molecular markers (nrITS, *trn*L-*trn*F, and *trn*H-*psb*A) for the new taxa and other Dominican and Haitian *Salvia* species to investigate their phylogenetic relationships.

## Abstract (es)

Describimos dos nuevas especies de *Salvia* provenientes de los bosques secos antillanos, pertenecientes a *Salvia* sección *Urbania*. Estas especies honran a dos botánicos latinoamericanos que han ampliado nuestro conocimiento ya sea sobre la flora dominicana o la familia de la menta (Lamiaceae). *Salviaclaseana* se encuentra en la Sierra Martín García. Se asemeja a *S.calaminthifolia* pero se diferencia de ella por tener tallos estrigosos, hojas rómbicas a truladas con una base cuneada, decurrente, y flores más grandes. *Salviamartineziana* habita en la Sierra de Bahoruco. Es semejante a *Salviabrachyphylla* pero difiere en tener tallos estrigosos con tricomas retrorsos y las flores dispuestas en las axilas de las hojas distales. Presentamos descripciones, fotografías, mapas de distribución y una clave de identificación para las especies de *Salvia* sección *Urbania* de República Dominicana. Además, secuenciamos tres marcadores moleculares (nrITS, *trn*L-*trn*F y *trn*H-*psb*A) para los nuevos taxones y otras especies de *Salvia* dominicanas y haitianas, con el fin de explorar sus relaciones filogenéticas.

## ﻿Introduction

The genus *Salvia* L. (sage) comprises more than 1000 species, which renders it the most diverse group in the mint family or Lamiaceae (Harley et al. 2004). Of these, more than half (580 spp.) belong to SalviaL.subgenusCalosphace (Benth.) Epling, a monophyletic group, that is endemic to the New World, distributed mainly in the Neotropics (González-Gallegos et al. 2020). The Antilles is one of the four diversity centers of Salviasubg.Calosphace, with ca. 45 species (Jenks et al. 2013). From the islands that form the Antilles, Hispaniola harbors the highest number of sages, with 36 species, 86% of them endemic (González-Gallegos et al. 2020).

Salviasubg.CalosphacesectionUrbania Epling is a group of 10 species endemic to Hispaniola, most of them distributed exclusively in Haiti (Epling 1939). These plants are characterized by a suffrutescent habit with intricate branching, small leaves, usually deltoid-ovate, obovate or flabelliform. The flowers grow in the leaf axils (1–3 per axil) or are rarely arranged in short racemes and are typically blue or violet in color. The calyx has 3-veined posterior lobes; the corolla is ventricose, invaginate and epapillate; the stamens are included in the galea, and the posterior stigmatic branch is longer than the anterior (Epling 1939).

Dominican Republic houses 21 species of *Salvia* (González-Gallegos et al. 2020), three of them from SalviasectionUrbania (Liogier 1994). During November of 2016, a botanical expedition was carried out in Dominican Republic, visiting the montane temperate and arid zones to collect different species of *Salvia*. Among the collected material, we found specimens of two species from section Urbania that do not match any of the currently described taxa. In this study we describe both and compare them to the taxa that morphologically resemble them the most. Additionally, we provide an identification key for the Dominican species of the section. Finally, using DNA sequences of three molecular markers (nrITS, *trn*H-*psb*A intergenic spacer and *trn*L-*trnF* region) from most of the sages from Dominican Republic and a few Haitian *Urbania* species, we evaluate the phylogenetic position of the new taxa and test the monophyly of section Urbania.

## ﻿Materials and methods

### ﻿Fieldwork, species identification and conservation status assessment

Herbarium specimens were collected and processed, and the individuals were photographed *in situ*. Voucher specimens of all the collected taxa were deposited at the herbarium of the Jardín Botánico Nacional Dr. Rafael M. Moscoso (JBSD) and the duplicates (when available) were sent to MEXU and XAL herbaria in Mexico (Thiers 2023). Additionally, for each of the herbarium specimens collected, leaf samples were taken for DNA extraction and flowers were preserved in alcohol for posterior morphological analysis.

The identification of the specimens was based on the most comprehensive revision of SalviasubgenusCalosphace by Epling (1939), the revision of *Salvia* of Hispaniola (Liogier 1994) and the revision of sections *Ekmania* (Torke 2000), *Gardoquiiflorae* (Zona et al. 2016) and *Wrightiana* (Zona et al. 2011).

The distribution map was made with QGIS v. 3.30 (QGIS Development Team, 2023), using the distribution data from the herbarium specimen and iNaturalist observations from one of the authors, these occurrences and coordinates are not provided due to the species global conservation status of Critically Endangered. The species’ extent of occurrence (EOO) and species’ area of occupancy (AOO) were calculated using GeoCAT (Bachman et al. 2011). Finally, the conservation status of the new taxa was assessed based on these results and considering the IUCN guidelines (2022).

### ﻿Taxon sampling, DNA extraction, amplification and sequencing

To test the phylogenetic position of the new taxa, as well as other sage species from the Caribbean, we sampled most of the species from Dominican Republic and a few taxa from Haiti, mainly belonging to section Urbania (Table 1; Suppl. material 1). DNA was extracted either from herbarium specimens or Silica gel-dried tissue, using the 2 × CTAB method (Doyle and Doyle 1987). DNA amplification followed the profiles and primer combinations described by Fragoso-Martínez et al. (2018), the only modification made is that the PCR reactions were carried out in 15 µL volumes. Finally, sequencing was performed at Macrogen, Inc.

**Table 1.** Voucher information and GenBank accession codes for the sampled sage species in this study. Detailed specimen information can be found in Suppl. material 1.

Species	Voucher information	GenBank accession numbers		
		ITS	*trn*H-*psb*A	*trn*L-*trn*F
*S.arborescens* Urb. & Ekman	*I. Fragoso-Martínez et al. 459* (JBSD, MEXU)	PP905439	PP907529	PP907549
*S.arduinervis* Urb. & Ekman	*E. Ekman 3168* (TEX)	PP905440	PP907530	PP907550
*S.bahorucona* Urb. & Ekman	*I. Fragoso-Martínez et al. 517* (JBSD, MEXU)	PP905441	PP907531	PP907551
*S.brachyloba* Urb.	*I. Fragoso-Martínez et al. 509* (JBSD, MEXU)	PP905442	PP907532	PP907552
*S.claseana Fragoso & Salazar	*I. Fragoso-Martínez et al. 529* (JBSD, MEXU)	PP905459	PP907548	PP907568
*S.calaminthifolia Vahl	*E. Ekman 9443* (TEX)	PP905443	PP907533	PP907553
*S.caymanensis* Millsp. & Uline	*I. Fragoso-Martínez & R. Middleton 309*, cultivated	PP905444	PP907534	PP907554
*S.decumbens* Alain	*I. Fragoso-Martínez et al. 519* (JBSD, MEXU)	PP905445	PP907535	PP907555
*S.foveolata* Urb. & Ekman	*I. Fragoso-Martínez et al. 508* (JBSD, MEXU)	PP905446	PP907536	PP907556
*S.lachnaioclada* Briq.	*I. Fragoso-Martínez et al. 608* (JBSD, MEXU)	PP905447	PP907537	PP907557
*S.lavendula* Alain	*T. Clase et al. 1059* (JBSD)	PP905448	PP907538	PP907558
*S.martineziana Fragoso & Martínez-Ambr.	*I. Fragoso-Martínez et al. 497* (JBSD, MEXU)	PP905458	PP907547	PP907567
*S.montecristina Urb. & Ekman	*I. Fragoso-Martínez et al. 527* (JBSD, MEXU)	PP905449	PP907539	PP907559
*S.praeterita Epling	*I. Fragoso-Martínez et al. 607* (JBSD, MEXU)	PP905450	PP907540	PP907560
*S.selleana* Urb.	*I. Fragoso-Martínez et al. 503* (JBSD, MEXU)	PP905451	PP907541	PP907561
*S.serotina* L.	*I. Fragoso-Martínez et al. 506* (JBSD, MEXU)	PP905452	PP907542	PP907562
*S.subaequalis Epling	GenBank BioSample: SAMN22547053	PP905453	–	–
*S.tenella* Sw.	*T. Clase et al. 8266* (JBSD)	PP905454	PP907543	PP907563
*S.thormanii* Urb.	*T. Clase et al. 8059* (JBSD)	PP905455	PP907544	PP907564
*S.tuerckheimii* Urb.	*I. Fragoso-Martínez et al. 607* (JBSD, MEXU)	PP905456	PP907545	PP907565
*S.uncinata* Urb.	*I. Fragoso-Martínez et al. 575* (JBSD, MEXU)	PP905457	PP907546	PP907566

*SalviasectionUrbania.

### ﻿Sequence edition and alignment

The sequences were edited and assembled using Geneious v.10.2.6 (http://www.geneious.com, Kearse et al. 2012). For one species (*S.subaequalis* Epling), we downloaded the raw data from the GenBank BioSample SAMN22547053 (Rose et al. 2021), and we assembled the ITS region using the default settings for the internal transcribed spacers in GetOrganelle (Jin et al. 2020). The edited and assembled sequences were uploaded to Genbank (www.ncbi.nlm.nih.gov/Genbank) with the accession numbers shown in Table 1. To determine the phylogenetic position of the new taxa, as well as other sage species from the Antilles, we combined the newly sequenced data with the most comprehensive data matrix of SalviasubgenusCalosphace from Fragoso-Martínez et al. (2018). Additionally, we included sequences from posterior studies of the same research group (González-Gallegos et al. 2018; Martínez-Ambriz et al. 2019; Fragoso-Martínez et al. 2021). The matrices of each marker were aligned individually, using MAFFT (Katoh and Standley 2013).

The matrix comprising the three molecular markers included 288 taxa, of which 269 belong to SalviasubgenusCalosphace. The length of this matrix was 2,197 bp: 722 bp from the nrITS region, 505 bp from the *trn*H-*psb*A intergenic spacer and 970 bp from the *trn*L-*trnF* region. Based on the results of the congruence test between plastid and nuclear data performed in previous phylogenetic studies of SalviasubgenusCalosphace (Fragoso-Martínez et al. 2018), and to include the most comprehensive dataset of the subgenus, we decided to concatenate the matrices and analyze them together. However, we are aware that a certain amount of discordance between these kinds of datasets is expected due to different phenomena (e.g., hybridization, introgression, chloroplast capture, etc.) or differences in the inheritance process between the plastid and nuclear DNA. Thus, the trees from the separate analyses (ITS and plastid) are provided as part of Suppl. material 2.

### ﻿Model selection and phylogenetic analyses

The concatenated matrix included three partitions corresponding to each molecular marker. For each partition, we evaluated 88 molecular substitution models using ModelFinder (Kalyaanamoorthy et al. 2017). The selected substitution models according to the Bayesian Information Criterion (BIC) were: GTR+F+I+G4 for the ITS region, TVM+F+I+G4 for the *trn*H-*psb*A IGS and TIM+F+G4 for the *trn*L-*trnF* region.

The Maximum likelihood analysis of the concatenated matrix was performed using the IQ-TREE algorithm (Nguyen et al. 2015) in the W-IQ-TREE web server (Trifinopoulos et al. 2016). Due to the length of the resulting tree, only the Angulatae clade is depicted in Fig. 4 and the complete tree is provided as Suppl. material 2. For this figure, the Angulatae clade was extracted from the main tree using the phytools package (Revell 2012) in R (R Core Team 2016) and edited with FigTree version 1.4.4 (Rambaut 2018).

## ﻿Results

### ﻿Taxonomic treatment

#### ﻿ Salvia claseana

Taxon classificationPlantaeLamialesLamiaceae

Fragoso & Salazar sp. nov.

07A5D4E1-A819-510F-AE53-8E3B01B25B73

urn:lsid:ipni.org:names:77352939-1

Fig. 1

##### Diagnosis.

Similar to *Salviacalaminthifolia*, but with the stems strigose (*vs.* cinereous); the leaves rhombic to trullate with the base cuneate, decurrent, the margin crenate-serrate (*vs.* deltoid-ovate, base truncate, margin subentire); the flowers bigger, with the calyx 5.5–8 mm long and the corolla tube 7.7–9 mm long (*vs.* 5–5.5 mm long and 5–6 mm long, respectively), and the lower lip reclinate (*vs.* reflexed).

**Figure 1.**
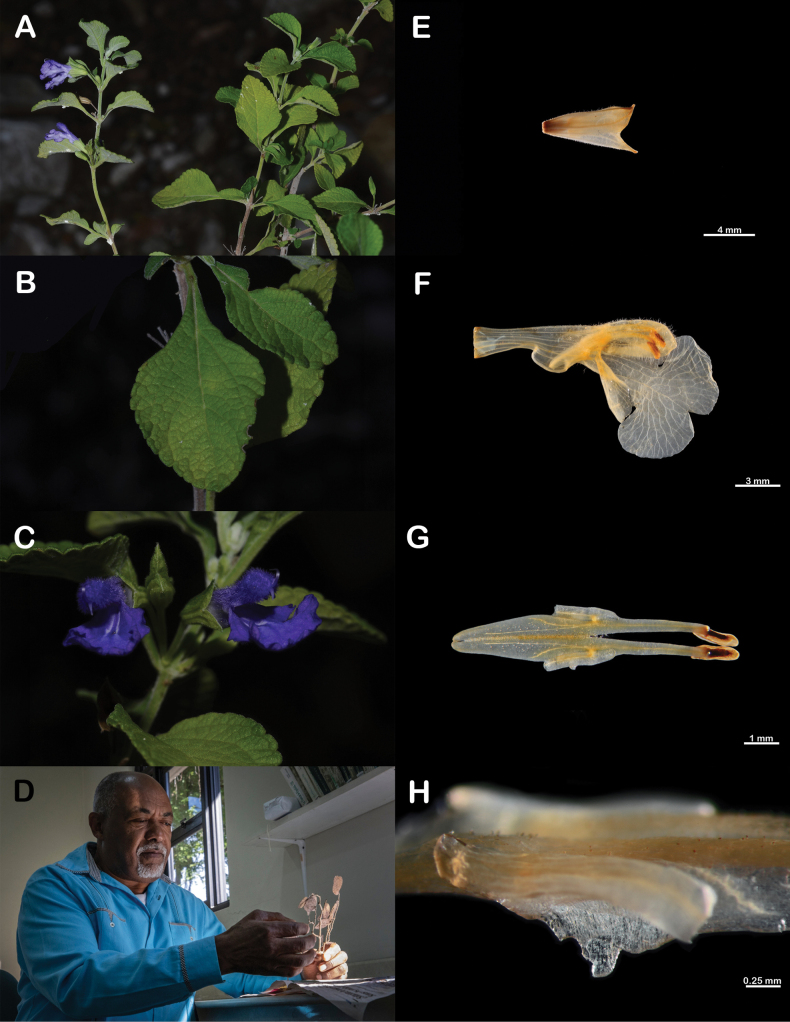
*Salviaclaseana* and the botanist that honors **A** flowering branch **B** leaves **C** flowers **D** Teodoro Clase at the JBSD herbarium **E** calyx **F** corolla **G** stamens **H** detail of the connective tooth (Specimen photographs taken from *I. Fragoso et al. 529*).

##### Type.

Dominican Republic. **Azua, Las Charcas** • Paraje Boquerón, cañada subiendo por el puente Juana Guayacán, a 4 km de la desviación de la carretera Sánchez (Bani-Azua); 18°21'0.9138"N, 70°31'51.8118"W; 307 m; 28 Nov 2016; *I. Fragoso-Martínez, G.A. Salazar & T. Clase 529* (holotype: JBSD 129667; isotypes: MEXU 1512146, XAL 0154233).

##### Description.

***Suffruticose herbs***, ca. 0.5 m tall; stems strigose with antrorsely appressed trichomes, internodes 1.5–4 cm long. ***Leaves*** rhombic to trullate, 1.3–3.6 × 1–3 cm, base cuneate, decurrent, apex acute, margin crenate-serrate, ciliate; upper leaf surface bullate, glabrescent; lower leaf surface pale, densely pubescent, simple trichomes minute, white, with amber spherical glands; petioles 0.5–1.2 cm long. ***Flowers*** axillary, 4(–8) per node; pedicels 2.5–6 mm long, trichomes simple, adpressed. ***Calyx*** green, tubular-campanulate, 5.5–8 mm long, strigose, with simple and glandular trichomes; tube 4.7–6.5 × 3 mm; lobes deltate, apex apiculate, upper lobe 1.3–1.8 mm long, straight, 3-veined, margin ciliate, lower lobes 1.3–2.5 mm long, straight. ***Corolla*** violet with white nectar guides in the lower lobe, ca. 1 cm long, tube 7.7–9 × 2.6–3 mm, ventricose, invaginate, internally epapillate; lobes unequal in length, upper lobe galeate, 3.8–6 mm long, densely pubescent, lower lobe 5–8 × 7.8–8.4 mm, tetralobate, reclinate. ***Stamens*** included in the upper corolla lobe, fused close to the corolla opening; filaments 1.5–2 mm long; connective 7–8 mm long, sparsely pilose, with a bilobed tooth close to the insertion with the filament, straight; upper arm of the connective shorter than the lower arm, 3–3.5 mm long, thecae 1–1.5 mm long; lower arm 4.5–5 mm long. ***Style*** 9–11 mm long, densely pubescent near the branches, upper branch longer than the lower one, lower branch spathulate. ***Nectary disc*** surface with spherical glands near the base of the mericarps, nectary horn ca. 2 mm long, oblong, laterally compressed. ***Mericarps*** ovoid, 1.6–2 × 0.7–0.9 mm, smooth.

##### Phenology.

Flowers were documented from November to May. Fruits have been observed after this period.

##### Etymology.

The epithet “*claseana*” honors the Dominican botanist Teodoro Clase, head of the Botany Department of the Jardín Botánico Nacional “Dr. Rafael M. Moscoso”. His botanical expeditions have resulted in ca. 12,000 collected specimens and he has described ca. 12 new species of angiosperms from Hispaniola. These contributions are undoubtedly crucial to the knowledge of the flora of Dominican Republic.

##### Distribution, habitat and conservation status.

Endemic to the dry forests with limestone soils from the Sierra Martín García in Azua, Dominican Republic (Fig. 3). *Salviaclaseana* is represented by three collections, each from a different population. In the GeoCat (Bachman et al. 2011) analysis, the species’ extent of occurrence (EEO) is 22.86 km^2^ with an area of occupancy (AOO) of 12 km^2^. Considering these results, combined with the high level of endemism of the region, and taking into account the IUCN criteria (IUCN, 2022), we suggest that *S.claseana* is placed in the category of Critically Endangered (CR).

##### Comments.

Phylogenetically, this species is closely related to *Salviapraeterita* (Fig. 4); however, the latter has the flowers arranged into racemes, while the new species produces flowers in the leaf axils. Morphologically, *Salviaclaseana* resembles *Salviacalaminthifolia* the most but differs from it by a number of characters (Table 2), from which the most conspicuous are: the pubescence, which is strigose in the former and cinereus in the latter, the shape and base of the leaves (rhombic to trullate, cuneate *vs.* deloid-ovate, truncate) and the size of its flowers (bigger in the new species, Table 2). Additionally, *Salviacalaminthifolia* is not closely related to the new species (Fig. 4) and it seems to be only distributed in Haiti (Liogier, 1994), despite the locality cited in the type specimen (Santo Domingo). No distribution data or collections from Dominican Republic were found representing this species either in the JBSD herbarium or global databases such as GBIF.org (GBIF Secretariat 2023).

**Table 2.** Morphological comparison among the new taxa and their more similar species, based data from type specimens, Epling (1939) and Liogier (1994).

	*S.claseana* Fragoso & Salazar	*S.calaminthifolia* Vahl	*S.praeterita* Epling	*S.martineziana* Fragoso & Martínez-Ambr.	*S.brachyphylla* Urb.
**STEM**					
**Pubescence**	Strigose, trichomes antrorse	Cinereous, trichomes straight	Strigose, trichomes antrorse	Strigose, trichomes retrorse	Hispid, trichomes straight
**LEAF**					
**Shape**	Rhombic to trullate	Deltoid-ovate	Ovate to deltoid-ovate	Obovate to flabellate	Obovate to flabellate
**Size (cm)**	1.3–3.6 × 1–3	0.5–1.2 × 0.7–1.5	1–2 × 0.5–1	1–1.5 × 0.5–1	1.5–3 × 1–2.5
**Base**	Cuneate, decurrent	Truncate	Cuneate, decurrent	Cuneate, decurrent	Cuneate, decurrent
**Margin**	Crenate-serrate	Subentire	Crenate-serrate	Crenate-serrate	Crenate-serrate
**Upper surface**	Bullate	Smooth	Bullate	Bullate	Bullate
**Lower surface**	Densely pubescent	Cinereous, trichomes adpressed	Densely pubescent	Tomentulose	Hispid
**INFLORESCENCE**					
**Presence**	Absent (axillary flowers)	Absent (axillary flowers)	Present, terminal racemes	Absent (axillary flowers)	Present, axillary and terminal racemes
**Flowers per node**	4(–8)	2–6	2–6	2	3
**CALYX**					
**Shape**	Tubular-campanulate	Tubular-campanulate	Tubular-campanulate	Campanulate	Tubular-campanulate
**Length (mm)**	5.5–8	5–5.5	5–7	5–6.8	5.5–6.5
**COROLLA**					
**Tube length (mm)**	7.7–9	5–6	8.5–9.5	7–9	7.5–9.5
**Upper lip length (mm)**	3.8–6	3–4.7	4–4.6	4.5–6.5	5.6–6
**Lower lip length (mm)**	5–8	3.5–5.5	5–6	7–7.5	ca. 6.6
**Lower lip position**	Reclinate	Reflexed	Reclinate	Reflexed	Reclinate

##### Additional specimens examined.

Dominican Republic. **Azua, Barreras** • Sierra Martín García, en los alrededores de Barreras; 170 m; 11 Sep 1984; *M. Mejía et al. 1180* (JBSD). **Las Charcas** • Parque Nacional Francisco Alberto Caamaño, paraje Boquerón; 18° 21'38.4366"N, 70°31'51.24"W; 298 m; 9 May 2014; *T. Clase & R. Ovidio S. 8645* (JBSD, MEXU).

#### ﻿ Salvia martineziana

Taxon classificationPlantaeLamialesLamiaceae

Fragoso & Martínez-Ambr. sp. nov.

297DA86E-FCCD-5F23-9674-B907F6D0CB64

urn:lsid:ipni.org:names:77352940-1

Fig. 2

##### Diagnosis.

Similar to *Salviabrachyphylla*, but differing from it by the pubescence of the plant (strigose with retrorse trichomes *vs.* hispid with erect trichomes); having smaller leaves (1–1.5 × 0.5–1 cm *vs.* 1.5–3 × 1–2.5) with a tomentulose lower leaf side (*vs.* hispid); flowers axillary (*vs.* forming racemes), calyx campanulate (*vs.* tubular-campanulate) and lower lobe of the corolla reflexed (*vs.* reclinate).

**Figure 2.**
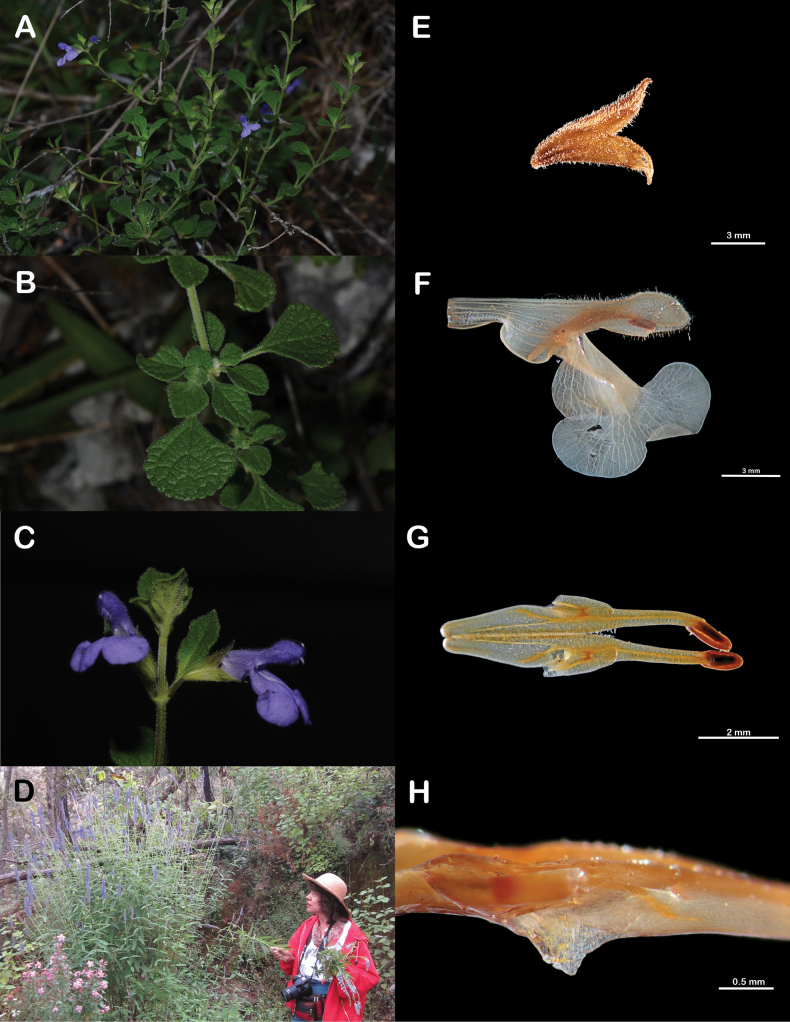
*Salviamartineziana* and the botanist that honors **A** flowering branch **B** leaves **C** flowers **D** Martha Martínez-Gordillo conducting fieldwork in Mexico **E** calyx **F** corolla **G** stamens **H** detail of the connective tooth (Specimen photographs taken from *I. Fragoso et al. 497*).

##### Type.

Dominican Republic. **Independencia, Duvergé** • Parque Nacional Sie­rra de Bahoruco, 6.8 km al S de Puerto Escondido por el camino a la Caseta 1; 18°17'10.4994"N, 71°34'6.3978"W; 965 m; 24 Nov 2016; *I. Fragoso-Martínez, G.A. Salazar & T. Clase 497* (holotype: JBSD 129573; isotypes: MEXU 1512142, XAL 0154234).

##### Description.

***Suffruticose herbs***, 0.3–0.5 m tall; stems strigose with retrorsely appressed trichomes, internodes 0.8–2.4 cm long. ***Leaves*** obovate to flabellate, 1–1.5 × 0.5–1 cm, base cuneate, decurrent, apex acute to obtuse, margin crenate-serrate, ciliate; upper leaf surface bullate, densely hirsute; lower leaf surface whitish, tomentulose simple trichomes minute, curved, white, with yellow spherical glands; petioles 4–8 mm long. ***Flowers*** axillary, 2 per node; pedicels 1.5–4.5 mm long, hirsute. ***Calyx*** green, campanulate, 5–6.8 mm long, densely hirsute, with simple and glandular trichomes; tube 3–5 × 3 mm; lobes ovate-deltate, apex apiculate, upper lobe 2.5–4 mm long, curved backwards, 3-veined, margin ciliate, lower lobes 1.6–2.5 mm long, straight. ***Corolla*** violet with white nectar guides in the lower lobe, 1.2–1.4 cm long, tube 7–9 × 2.9 mm, ventricose, invaginate, internally epapillate, lobes unequal in length, upper lobe galeate, 4.5–6.5 mm long, densely pubescent, lower lobe 7–7.5 × 8.4 mm, tetralobate, reflexed. ***Stamens*** included in the upper corolla lobe, fused close to the corolla opening; filaments 1.5–2 mm long; connective 7.5–8.5 mm long, sparsely pilose, with an entire tooth close to the insertion with the filament, retrorse; upper arm of the connective slightly longer than the lower arm, 4–4.5 mm long, thecae 1–1.5 mm long; lower arm 3.5–4 mm long. ***Style*** 13–15 mm long, densely pubescent near the branches, with simple and capitate glandular trichomes, upper branch longer than the lower one, lower branch spathulate. ***Nectary disc*** surface with spherical glands near the base of the mericarps, nectary horn ca. 1 mm long, oblong, laterally compressed. ***Mericarps*** ovoid, 1.5–2 × 0.4–0.6 mm, smooth.

##### Phenology.

Flowers were documented from November to May. Fruits have been observed after this period.

##### Etymology.

The epithet “*martineziana*” honors the Mexican botanist Martha Martínez Gordillo, specialist of the Euphorbiaceae and Lamiaceae families. Dr. Martínez works at the FCME herbarium at the Universidad Nacional Autónoma de México. She has conducted fieldwork mainly in the states of Chiapas, Guerrero, Mexico and Oaxaca. Her study of the Mexican flora, particularly that of Gue­rrero, has led to the description of more than 30 species of angiosperms, many of them from the genus *Salvia*. Dr. Martínez has taught botany to numerous generations of Mexican biologists, and her exemplary professional and academic ethics, determination, kindness and generosity are an inspiration to her students.

##### Distribution, habitat and conservation status.

**Figure 3.**
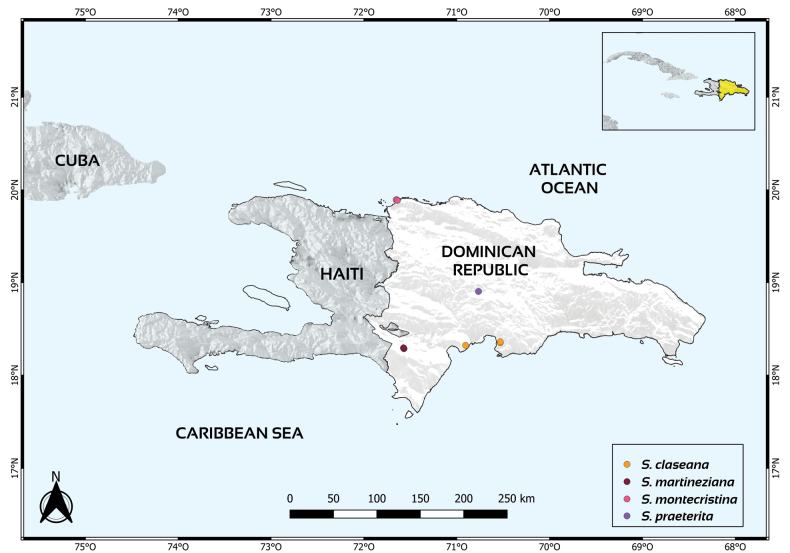
Distribution map of the four taxa of SalviasectionUrbania from Dominican Republic. *Salviacalaminthifolia* was excluded from the map due to the lack of distribution data in the country aside from the type specimen.

Endemic to the dry forests with limestone soils from Sierra de Bahoruco in Independencia, Dominical Republic (Fig. 3). *Salviamartineziana* is represented by two collections, each seemingly belonging to different populations, both from the southern region of Puerto Escondido in the Sierra de Barohuco. In the GeoCat (Bachman et al. 2011) analysis, the species’ extent of occurrence (EEO) is 0.09 km^2^ with an area of occupancy (AOO) of 8 km^2^. Considering these results, combined with the high level of endemism of the region, and taking into account the IUCN criteria (IUCN 2022), we suggest that *S.martineziana* should be placed in the category of Critically Endangered (CR).

##### Comments.

**Figure 4.**
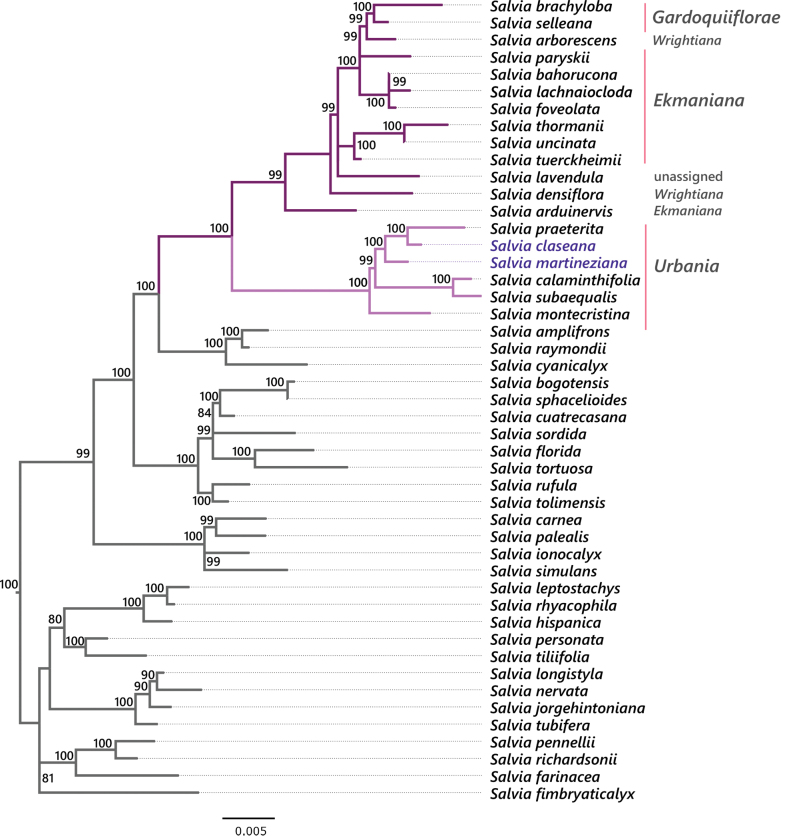
Phylogenetic relationships of the Angulatae clade based on three molecular markers (nrITS, *trn*H-*psb*A and *trn*L-*trn*F) with an increased taxon sampling including the new species and other Hispaniolan taxa. Bootstrap values ≥80% are shown above the branches. The names of the new species are written in purple, while the names in gray correspond to the sections of the taxa from the Hispaniolan subclade. An extended version of the phylogenetic tree is provided in Suppl. material 2.

This species is sister to a clade formed by two other Dominican species of SalviasectionUrbania (Fig. 4). It differs from *S.praeterita* by lacking flowers in racemes and from *S.claseana* by the size of the leaves (smaller in *S.martineziana*: 1–1.5 × 0.5–1 cm *vs.* 1.3–3.6 × 1–3 cm) and the pubescence of the lower side of the blades (tomentulose *vs.* densely pubescent). Morphologically, *S.martineziana* resembles *S.brachyphylla* the most, a Haitian species. However, it differs mainly by the lack of racemes, having the flowers distributed in the axils of the upper portion of the branches.

##### Additional specimens examined.

Dominican Republic. **Independencia, Duvergé** • 5.2 km al S de Puerto Escondido en camino a Acetillar, Sierra de Bahoruco; 170 m; 9 May 1985; *T. Zanoni et al. 34648* (JBSD).

### ﻿Identification key for the species of SalviasectionUrbania

**Table:** 

1a	Flowers in racemes	**2**
2a	Leaves and calyces densely hispid	***S.brachyphylla***
2b	Leaves and calyces strigose to glabrate	**3**
3a	Bracts persistent	**4**
4a	Bracts flabellate, surrounding the flowers almost completely	***S.saccifera***
4b	Bracts ovate, not surrounding the flowers completely	***S.subglabra***
3b	Bracts deciduous	**5**
5a	Lower leaf surface greenish, glabrous; flowers 6–12 per node	***S.hotteana***
5b	Lower leaf surface whitish, strigose; flowers 2–6 per node	***S.praeterita***
1b	Flowers axillary, not forming racemes	**6**
6a	Leaf margin subentire, entire or sinuate	**7**
7a	Leaves coriaceous	***S.montecristina***
7b	Leaves membranaceus	**8**
8a	Lower leaf surface incanous, grayish	***S.mornicola***
8b	Lower leaf surface glabrate, greenish	**9**
9a	Leaves oblong-elliptical	***S.tortuensis***
9b	Leaves deltoid-ovate	***S.subaequalis***
6b	Leaf margin crenate-serrate	**10**
10a	Leaf base truncate	***S.calaminthifolia***
10b	Leaf base cuneate	***11***
11a	Leaves rhombic to trullate, 1–3 cm wide, lower leaf surface densely pubescent	***S.claseana***
11b	Leaves obovate to flabellate, 0.5–1 cm wide, lower leaf surface tomentulose	***S.martineziana***

### ﻿Phylogenetic relationships

The Hispaniolan species included in the phylogenetic analysis are distributed in four different clades. The species from SalviasectionMicranthae (Benth.) Epling — *Salviaserotina* L. and *S.tenella* Sw.—, are part of a clade that also contains taxa from sections *Bracteata* Epling and *Subrotundae* (Epling) Epling (99% BS; Suppl. material 1). *Salviadecumbens* Alain is included in the Flocculosae clade, along with species from the Andean region such as *S.discolor* Kunth and S. *leucocephala* Kunth (100% BS; Suppl. material 1). *Salviaoccidentalis* Sw. is part of the Uliginosae clade and it is closely related to other species of section Microsphace (Briq.) Benth. (100% BS; Suppl. material 1). Most of the taxa surveyed are found in the Angulatae clade (Fig. 4), forming a Hispaniolan subclade (100% Bootstrap Support; BS). The species from this subclade belong to four different sections (Table 3) and one is unassigned (*Salvialavendula* Alain). From these, only sections *Urbania* Epling and *Gardoquiiflorae* Epling are monophyletic (both with 100% BS). However, the latter is nested in a clade formed by species of two sections: the paraphyletic *Ekmania* Epling and the polyphyletic *Wrightiana* (Fig. 4).

**Table 3.** Sections sampled and the percentage of taxa sequenced in this study.

Epling’s sections sampled	Taxa sampled from the total
*Ekmania* Epling	8 spp. (100%)
*Gardoquiiflorae Epling	2 spp. (66%)
*Micranthae*	4 spp. (57%)
*Urbania Epling	6 spp. (50%)
*Wrightiana* Epling	2 spp. (66%)

* Monophyletic sections.

The new species described in the present study belong to the monophyletic section Urbania. Within this clade *Salviamontecristina* Urb. & Ekman, endemic to Dominican Republic, is sister to the remaining species (Fig. 4). These taxa are arranged into two subclades, one containing Haitian species (100% BS) and another one formed by Dominican taxa (99% BS). The latter subclade comprises the new species and *Salviapraeterita* Epling.

## ﻿Discussion

The new taxa described here are an addition to the sage species of Dominican Republic, increasing its diversity to 23 species (González-Gallegos et al. 2020), five of them belonging to SalviasectionUrbania. As depicted in Fig. 3, these taxa are microendemic and their range of distribution is very narrow. Consequently, the conservation status assessment for the new taxa placed them both in the critically endangered (CR) category, following the IUCN criteria (IUCN 2022). Future studies should address the state and characteristics of the populations, to provide further insight into their dynamics and expand information about their conservation status.

Both species described here fit the circumscription of SalviasectionUrbania provided by Epling (1939). Moreover, this section was proved here to be monophyletic, rendering its phylogenetic relationships congruent with the morphology of the group. The clade formed by this section is related to other Hispaniolan taxa belonging to three other sections, only one of them monophyletic (Table 3). These results are consistent with the findings of Fragoso-Martínez et al. (2018), where many of the clades of the Neotropical sages show strong geographical structure instead of morphological congruence. Nevertheless, section Urbania shows both morphological and geographic structures. Only a few of the 41 non-monotypic sections sampled to date show the same pattern (e.g., *Lavanduloideae* Epling, *Membranaceae* (Benth.) Epling and *Sigmoideae* Epling); however, less than half of the species of SalviasubgenusCalosphace have been sequenced. Thus, there are still relationships to be explored or defined.

Zona et al. (2016) conducted the first phylogenetic analysis of Hispaniolan sages. Their study, based on five species, suggested that the island’s sage diversity originated from at least two colonization events. Our expanded taxonomic sampling has now revealed two additional colonization events: one involving species from section Micranthae and another from section Microsphace.

Despite a recent surge in the discovery of new Neotropical sages, the Dominican Republic has remained relatively unexplored in this regard. The last new sage species from the country was described almost 40 years ago (Liogier 1988). This underscores the critical need for continued botanical expeditions and taxonomic research to broaden our understanding of plant diversity in this region. Furthermore, incorporating more species of SalviasubgenusCalosphace into phylogenetic analyses will provide valuable insights into the evolutionary history of this lineage, and will allow us to group taxa with their closest relatives.

These newly discovered taxa from Hispaniola, which are critically endangered, are the first species of SalviasectionUrbania to be evaluated under the IUCN criteria. However, they mirror a pattern seen in other native sages on the island, where some species are vulnerable (*Salviaarborescens* Urb. & Ekman, and *S.decumbens* Alain) and others are critically endangered (*Salviabuchii* Urb., *S.haitiensis* Urb., *S.lachnaioclada* Briq. and *S.paryskii* Skean & Judd). These results highlight the need for a comprehensive assessment of all Hispaniolan sage species, to develop effective strategies for their protection.

## Supplementary Material

XML Treatment for Salvia claseana

XML Treatment for Salvia martineziana

## ﻿Additional information

### Author ORCIDs

Itzi Fragoso-Martínez https://orcid.org/0000-0003-3661-1076

Gerardo A. Salazar https://orcid.org/0000-0002-5203-5374

Emmanuel Martínez-Ambriz https://orcid.org/0000-0002-4770-4174

Martin Reith https://orcid.org/0000-0001-7830-9161

### Data availability

All the newly sequenced data was uploaded to the NCBI with the accession numbers provided in Table 1. The complete phylogenetic tree of the combined dataset is provided as Suppl. material 2.

## Supplementary materials

Supplementary material 1 Additional information of the sampled sage species in the study Data type pdf https://binary.pensoft.net/file/1191256This dataset is made available under the Open Database License (http://opendatacommons.org/licenses/odbl/1.0/). The Open Database License (ODbL) is a license agreement intended to allow users to freely share, modify, and use this Dataset while maintaining this same freedom for others, provided that the original source and author(s) are credited.Itzi Fragoso-Martínez, Gerardo A. Salazar, Emmanuel Martínez-Ambriz, Martin Reith

Supplementary material 2 Complete phylogenetic trees of the plastid, nuclear and combined dataset Data type pdf https://binary.pensoft.net/file/1191257This dataset is made available under the Open Database License (http://opendatacommons.org/licenses/odbl/1.0/). The Open Database License (ODbL) is a license agreement intended to allow users to freely share, modify, and use this Dataset while maintaining this same freedom for others, provided that the original source and author(s) are credited.Itzi Fragoso-Martínez, Gerardo A. Salazar, Emmanuel Martínez-Ambriz, Martin Reith

## References

[B1] Bachman SP Moat J Hill A de la Torre J Scott B (2011) Supporting Red List threat assessments with GeoCAT: Geospatial conservation assessment tool. ZooKeys 28: 117–126. 10.3897/zookeys.150.2109 PMC3234434 22207809

[B2] Doyle J Doyle J (1987) A rapid DNA isolation procedure for small quantities of fresh leaf tissue. Phytochemical Bulletin 19: 11–15.

[B3] Epling C (1939) A revision of SalviasubgenusCalosphace. Repertorium Specierum Novarum Regni Vegetabilis 110: 380.

[B4] Fragoso-Martínez I Martínez-Gordillo M Salazar GA Sazatornil F Jenks AA García Peña MR Barrera-Aveleida G Benitez-Vieyra S Magallón S Cornejo-Tenorio G Granados Mendoza C (2018) Phylogeny of the Neotropical sages (Salviasubg.Calosphace; Lamiaceae) and insights into pollinator and area shifts. Plant Systematics and Evolution 304(1): 43–55. 10.1007/s00606-017-1445-4

[B5] Fragoso-Martínez I Martínez-Gordillo M Salas S (2021) *Salviafimbriaticalyx*, a new species of *Salvia* (Lamiaceae) from Oaxaca, Mexico. Phytotaxa 518: 241–250. 10.11646/phytotaxa.518.4.1

[B6] GBIF Secretariat (2023) *Salviacalaminthifolia* Vahl. GBIF Backbone Taxonomy. Checklist dataset. https://www.gbif.org/es/species/3882857 [accessed: 04.08.2024]

[B7] González-Gallegos JG Fragoso-Martínez I González-Adame G Martínez-Ambriz E López-Enríquez I (2018) *Salviaozolotepecensis*, *S.patriciae* and *S.sirenis* (Lamiaceae), three new species from Miahuatlán district, Oaxaca, Mexico. Phytotaxa 352(2): 143–159. 10.11646/phytotaxa.362.2.2

[B8] González-Gallegos JG Bedolla-García BY Cornejo-Tenorio G Fernández-Alonso JL Fragoso-Martínez I García-Peña MR Harley RM Klitgaard B Martínez-Gordillo MJ Wood JRI Zamudio S Zona S Xifreda CC (2020) Richness and distribution of SalviasubgenusCalosphace (Lamiaceae). International Journal of Plant Sciences 181(8): 831–856. 10.1086/709133

[B9] Harley RM Atkins S Budantsev AL Cantino PD Conn BJ Grayer R Harley MM de Kok R Krestovskaja T Morales R Paton AJ Ryding O Upson T (2004) Lamiaceae. In: Kubitzki K, Kadereit JW (Eds) The families and genera of Vascular Plants VII. Dicotyledons: Lamiales (except Acanthaceae including Avicenniaceae). Springer, Berlin, 167−275. 10.1007/978-3-642-18617-2_11

[B10] IUCN (2022) Guidelines for Using the IUCN Red List Categories and Criteria. Ver. 15.1. Prepared by the Standards and Petitions Committee. https://www.iucnredlist.org/documents/RedListGuidelines.pdf

[B11] Jenks AA Walker JB Kim S-C (2013) Phylogeny of New World SalviasubgenusCalosphace (Lamiaceae) based on cpDNA (*psb*A-*trn*H) and nrDNA (ITS) sequence data. Journal of Plant Research 126(4): 483–496. 10.1007/s10265-012-0543-1 23263465

[B12] Jin J-J Yu W-B Yang J-B Song Y dePamphilis CW Yi T-S Li D-Z (2020) GetOrganelle: A fast and versatile toolkit for accurate de novo assembly of organelle genomes. Genome Biology 21(1): 241. 10.1186/s13059-020-02154-5 32912315 PMC7488116

[B13] Kalyaanamoorthy S Minh BQ Wong TKF Von Haeseler A Jermiin LS (2017) ModelFinder: Fast model selection for accurate phylogenetic estimates. Nature Methods 14(6): 587–589. 10.1038/nmeth.4285 28481363 PMC5453245

[B14] Katoh K Standley DM (2013) MAFFT multiple sequence alignment software version 7: Improvements in performance and usability. Molecular Biology and Evolution 30(4): 772–780. 10.1093/molbev/mst010 23329690 PMC3603318

[B15] Kearse M Moir R Wilson A Stones-Havas S Cheung M Sturrock S Buxton S Cooper A Markowitz S Duran C Thierer T Ashton B Meintjes P Drummond A (2012) Geneious Basic: An integrated and extendable desktop software platform for the organization and analysis of sequence data. Bioinformatics (Oxford, England) 28(12): 1647–1649. 10.1093/bioinformatics/bts199 22543367 PMC3371832

[B16] Liogier AH (1988) Novitates Antillanae XIV. Phytologia 64(5): 345–348.

[B17] Liogier AH (1994) *Salvia* (Labiatae). In: Liogier AH (Ed.) La Flora de La Española VI.Universidad Central del Este, San Pedro de Macorís, 293–314.

[B18] Martínez-Ambriz E Fragoso-Martínez I Martínez-Gordillo M (2019) A new species of *Salvia* from the *Fulgentes* clade (Lamiaceae), from Puebla, Mexico. Phytotaxa 409(1): 29–38. 10.11646/phytotaxa.409.1.4

[B19] Nguyen L-T Schmidt HA von Haeseler A Minh BQ (2015) IQ-TREE: A fast and effective stochastic algorithm for estimating maximum-likelihood phylogenies. Molecular Biology and Evolution 32(1): 268–274. 10.1093/molbev/msu300 25371430 PMC4271533

[B20] QGIS Development Team (2023) QGIS Geographic Information System Ver. 3.30. Open Source Geospatial Foundation Project. http://qgis.osgeo.org [accessed: 01.08.2024]

[B21] R Core Team (2016) R: A language and environment for statistical computing. R Foundation for Statistical Computing, Vienna. https://www.R-project.org/

[B22] Rambaut A (2018) FigTree. version 1.4.4. http://tree.bio.ed.ac.uk/software/figtree/ [accessed: 5.07.2020]

[B23] Revell LJ (2012) phytools: An R package for phylogenetic comparative biology (and other things). Methods in Ecology and Evolution 3(2): 217–223. 10.1111/j.2041-210X.2011.00169.x

[B24] Rose JP Kriebel R Kahan L DiNicola A González-Gallegos JG Celep F Lemmon EM Lemmon AR Sytsma KJ Drew BT (2021) Sage insights into the phylogeny of *Salvia*: Dealing with sources of discordance within and across genomes. Frontiers in Plant Science 12: 1–14. 10.3389/fpls.2021.767478 PMC8652245 34899789

[B25] Thiers B (2023) Index Herbariorum: a global directory of public herbaria and associated staff. New York Botanical Garden’s Virtual Herbarium. http://sweetgum.nybg.org/ih [accessed: 20.07.2024]

[B26] Torke B (2000) A revision of Salviasect.Ekmania (Lamiaceae). Brittonia 52: 265–302. 10.2307/2666577 [February 19, 2014]

[B27] Trifinopoulos J Nguyen L-T von Haeseler A Minh BQ (2016) W-IQ-TREE: A fast online phylogenetic tool for maximum likelihood analysis. Nucleic Acids Research 1–4(W1): W232–W235. 10.1093/nar/gkw256 PMC4987875 27084950

[B28] Zona S Clase T Franck A (2011) A Synopsis of SalviaSectionWrightiana (Lamiaceae). Harvard Papers in Botany 16: 383–388. 10.3100/0.25.016.0208

[B29] Zona S Finch K Clase T Jestrow B (2016) A synopsis of Salviasect.Gardoquiiflorae (Lamiaceae), with a note on the origins of Caribbean *Salvia* species. Phytotaxa 255(3): 214–226. 10.11646/phytotaxa.255.3.3

