# Supplementary material for: ﻿Two new species of Salvia (Lamiaceae) from the dry forests of Dominican Republic
Source: PhytoKeys. 2024 Dec 5;249:299–315. doi: 10.3897/phytokeys.249.137556 (PMC11638711; doi:10.3897/phytokeys.249.137556)
Supplement: Supplementary material 1 — Additional information of the sampled sage species in the study [file phytokeys-249-299_article-137556__-s001.pdf]

1. ***S. arborescens* Urb. & Ekman**

DOMINICAN REPUBLIC. Barahona, Polo: Sierra de Bahoruco, Los Arroyos de Polo, por la carretera que va de Polo a Enriquillo, *I. Fragoso-Martínez, G.A. Salazar & T. Clase 459* (JBSD, MEXU).

2. ***S. arduinervis* Urb. & Ekman**

HAITI. Massif de la Selle: Morne de la Selle, *E. Ekman 3168* (TEX).

3. ***S. bahorucona* Urb. & Ekman**

DOMINICAN REPUBLIC. Pedernales, Pedernales: Parque Nacional Sierra de Bahoruco, Comunidad Las Mercedes, km 28 carr. Panorámica Cabo Rojo-Aceitillar (entre la caseta y el Hoyo de Pelempito), *I. Fragoso-Martínez, G.A. Salazar & T. Clase 517* (JBSD, MEXU).

4. ***S. brachyloba* Urb.**

DOMINICAN REPUBLIC. Pedernales, Pedernales: Parque Nacional Sierra de Bahoruco, 500 m antes del puesto militar del Aguacate, *I. Fragoso-Martínez, G.A. Salazar & T. Clase 509* (JBSD, MEXU).

5. ***S. claseana* Fragoso & Salazar**

DOMINICAN REPUBLIC. Azua, Las Charcas: Paraje Boquerón, cañada subiendo por el puente Juana Guayacán, a 4 km de la desviación de la carretera Sánchez (Bani-Azua), *I. Fragoso-Martínez, G.A. Salazar & T. Clase 529* (JBSD, MEXU).

6. ***S. calaminthifolia* Vahl**

HAITI. Massif du Nord: Les Gonaïves, *E. Ekman 9443* (TEX).

7. ***S. decumbens* Alain**

DOMINICAN REPUBLIC. Pedernales, Pedernales: Parque Nacional Sierra de Bahoruco, Hoyo de Pelempito, borde del precipicio, *I. Fragoso-Martínez, G.A. Salazar & T. Clase 519* (JBSD, MEXU).

8. *S. foveolata* Urb. & Ekman

DOMINICAN REPUBLIC. Pedernales, Pedernales: Parque Nacional Sierra de Bahoruco, 4.8 km al NE del Puesto Militar los Arroyos, por la carretera internacional, *I. Fragoso-Martínez, G.A. Salazar & T. Clase 508* (JBSD, MEXU).

9. *S. lachnaioclada* Briq.

DOMINICAN REPUBLIC. Distrito Nacional, Santo Domingo: Cultivated plant at the Jardín Botánico José María Moscoso, *I. Fragoso-Martínez, G.A. Salazar & T. Clase 608* (JBSD, MEXU).

10. *S. lavendula* Alain

DOMINICAN REPUBLIC. Santiago, San José de las Matas: Cordillera Central, sección Mata Grande, en montes Prietos, cabecera del Río Bao, Parque A. Bermúdez, T. *Clase et al. 1059* (JBSD).

11. *Salvia martineziana* Fragoso & Martínez-Ambr.

DOMINICAN REPUBLIC. Independencia, Duvergé: Parque Nacional Sierra de Bahoruco, 6.8 km al S de Puerto Escondido por el camino a la Caseta 1, *I. Fragoso-Martínez, G.A. Salazar & T. Clase 497* (JBSD, MEXU, XAL).

12. *S. montecristina* Urb. & Ekman

DOMINICAN REPUBLIC. Distrito Nacional, Santo Domingo: Planta cultivada en el Jardín Botánico José María Moscoso, *I. Fragoso-Martínez, G.A. Salazar & T. Clase 527* (JBSD, MEXU).

13. *S. praeterita* Epling

DOMINICAN REPUBLIC. Distrito Nacional, Santo Domingo: Planta cultivada en el Jardín Botánico José María Moscoso, *I. Fragoso-Martínez, G.A. Salazar & T. Clase 607* (JBSD, MEXU).

14. ***S. selleana*** **Urb.**

DOMINICAN REPUBLIC. Independencia, Duvergé: Parque Nacional Sierra de Bahoruco, paraje ca. 1.5 km pasando la Caseta 2, *I. Fragoso-Martínez, G.A. Salazar & T. Clase 503* (JBSD, MEXU).

15. ***S. serotina*** **L.**

DOMINICAN REPUBLIC. Pedernales, Pedernales: La Manigua, carr. Internacional subiendo hacia la comunidad Los Arroyos, cerca de la frontera con Haití, *I. Fragoso-Martínez, G.A. Salazar & T. Clase 506* (JBSD, MEXU).

16. ***S. tenella*** **Sw.**

DOMINICAN REPUBLIC. Barahona, Enriquillo: Sierra de Bahoruco, en la Isleta por la carretera hacia la Matereza en finca del Sr. Paquito, *T. Clase et al. 8266* (JBSD).

17. ***S. thormanii*** **Urb.**

DOMINICAN REPUBLIC. San José de Ocoa, El Pinar: subiendo desde El Palmarito hacia el Montazo, orilla del camino, *T. Clase et al. 8059* (JBSD).

18. ***S. tuerckheimii*** **Urb.**

DOMINICAN REPUBLIC. La Vega, Constanza: 4.68 km al W de Constanza rumbo al Pueblo de Guanito, pasando el Río Arroyo Hondo, *I. Fragoso-Martínez, G.A. Salazar & T. Clase 603* (JBSD, MEXU).

19. ***S. uncinata*** **Urb.**

DOMINICAN REPUBLIC. San José de Ocoa, Sabana Larga: Pajón Blanco, cerca de la bifurcación a la antena de control de incendios, 30 km al S de Constanza, *I. Fragoso-Martínez, G.A. Salazar & T. Clase 575* (JBSD, MEXU).
