# Supplementary material for: ﻿Two new species of Salvia (Lamiaceae) from the dry forests of Dominican Republic
Source: PhytoKeys. 2024 Dec 5;249:299–315. doi: 10.3897/phytokeys.249.137556 (PMC11638711; doi:10.3897/phytokeys.249.137556)

Supplementary material 2.

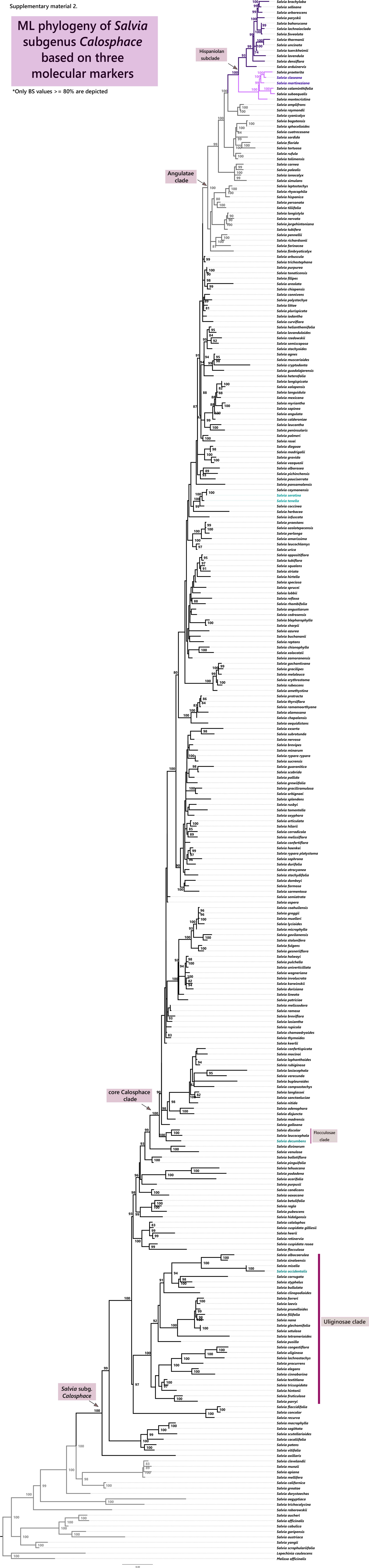

Supplementary material 2.

ML phylogeny of *Salvia* subgenus *Calosphace* based on nrITS

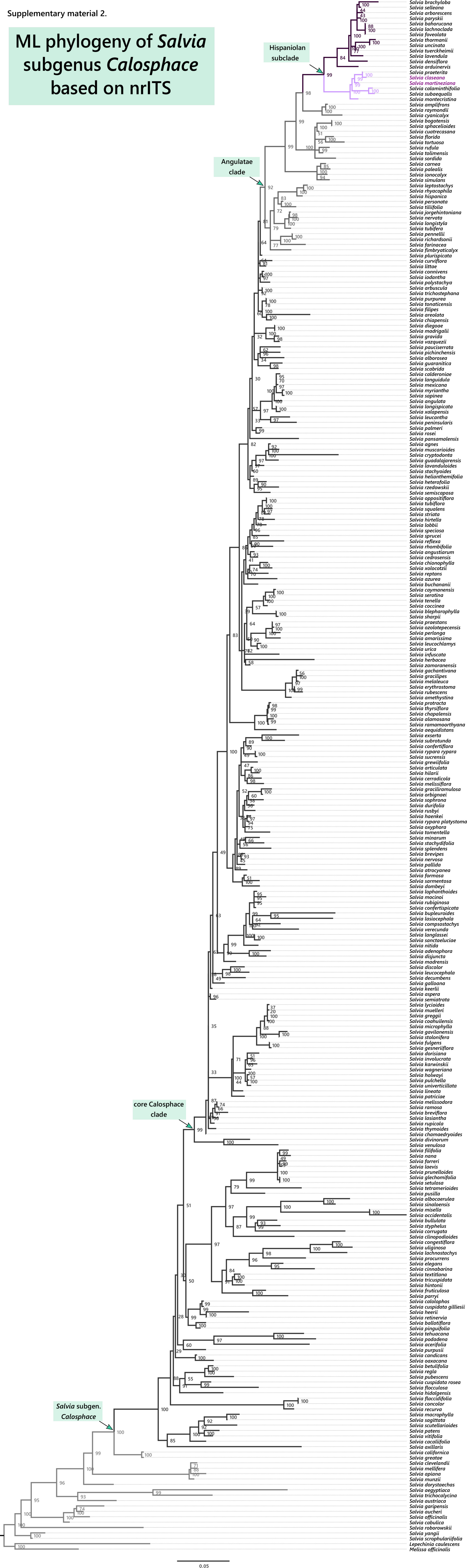

Supplementary material 2.

ML phylogeny of *Salvia* subgenus *Calosphace* based on plastid data

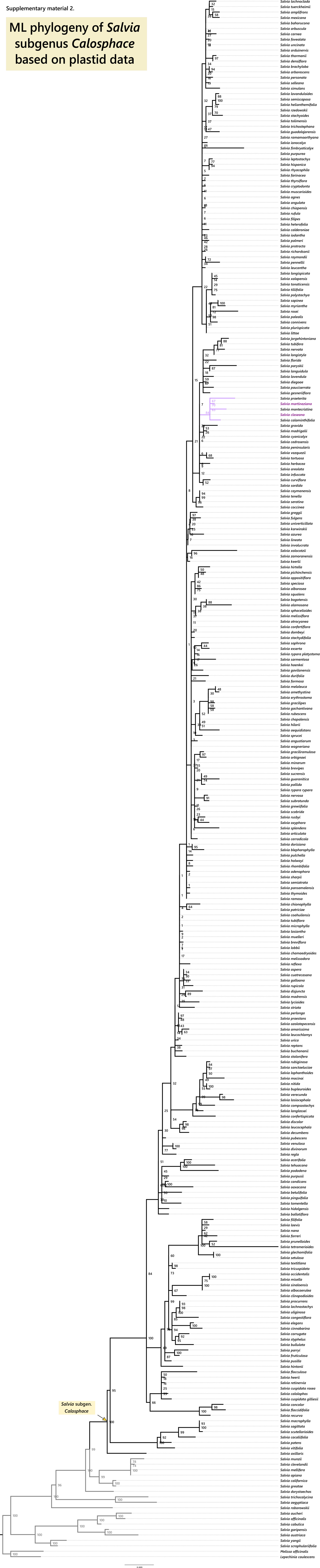

Supplement: Supplementary material 2 — Complete phylogenetic trees of the plastid, nuclear and combined dataset [file phytokeys-249-299_article-137556__-s002.pdf]
